# Progress in Medicine

**Published:** 1900-04-14

**Authors:** 


### Progress in Medicine.

### DISEASES OF THE DIGESTIVE SYSTEM.

**Chemical Tests.**—Nitrohydroxylamin may be used as a test for free hydrochloric acid in stomach contents. Organic acids, such as lactic and acetic, and acid phosphates, do not decompose this substance, which in the presence of hydrochloric acid breaks up, with evolution of nitrogen dioxide, from the estimate of which the amount of free H.Cl. may be inferred.[1]

The motor activity of the stomach[2] may be estimated by iodipin. Under normal conditions this substance is passed unaltered through the stomach, and is broken up by the pancreatic juice and bile, with liberation of iodine, which appears in the urine in twenty minutes, and in the saliva in half-an-hour. The appearance of the iodine is tested with starch-paper, and if the periods mentioned above are exceeded, it may be inferred that the motor-function is impaired. Iodipin is a combination of iodine with oil of sesamum.

**Gastric Lavage.** — Williams[3] recommends gastric lavage not only in dilated stomach conditions, but also in acute gastritis, acute fermentative dyspepsia, chronic gastritis, and nervous dyspepsia, the operation helping to stimulate the circulation and increase glandular activity and peristalsis. Mucus is more easily removed if a tablespoonful of salt or bicarbonate of soda be added to each quart of water. He prefers to wash out the stomach after a light breakfast, passing in a pint to a quart of fluid each time. The stomach-tube may also be used for the purpose of procuring stomach contents for chemical analysis, the patient being directed to sharply contract the abdominal muscles directly the tube reaches the stomach, in this way expelling a quantity of its contents. The objection to the simple funnel and syphon for gastric lavage is, that syphonic action has to be started afresh after each introduction of fluid. The use of the Leube Rosenthal apparatus, with its -shaped piece, obviates this, but it is too complicated for use in general practice. Herschell has recently devised a three-way tap by means of which continuous lavage may be maintained. The London agents for this invention are Messrs. Allen and Hanburys.

Previous to washing out the stomach, and with a view to mapping out its exact dimensions, it has been customary to administer effervescent powders, the gas from which distends and defines the outline of the organ. Fürbringer and Krönig[4] point out that this proceeding is not altogether devoid of discomfort and danger to the patient, and that a more certain and efficacious way of introducing air into the organ is by passing a stomach-tube into the upper part of the gullet, and then blowing in air either by the mouth or by a pair of bellows made for the purpose. So tightly gripped is the tube that no air regurgitates back into the pharynx.

**Gastric Ulcer.**—During the years 1888-1898, 187 cases of gastric ulcer were treated at the Massachusetts General Hospital. Reviewing these cases, Greenhalgh and Joslin[5] find vomiting to be a constant symptom, being only absent in four cases. Its time of occurrence was very variable. Pain was present in 173 cases, being most frequent in the epigastrium, and seldom extending to the right of the middle line. In some severe and fatal cases no pain was felt. Hæmorrhage was present in 147 cases, and terminated fatally in 7, the majority of whom were men. Six cases perforated. The disease is far more fatal in men (30 per cent.) than in women (9 per cent.), but 64 per cent. of sufferers are cured, 118 per cent. temporarily relieved. Saundby[6] estimates that the proportion of women to men suffering from gastric ulcer is 20 to 1. Most cases occur in anæmic subjects, between the ages of 18 and 38. The mortality amongst treated cases is about 5 per cent. The typical symptoms are: Pain soon after food, relieved by vomiting, hæmatemesis, tenderness in epigastrium, and constipapation. The supply of pepsine is normal, the motor function of the stomach uninfluenced, hydrochloric acid may be in excess or diminished. The disease is differentiated from anæmic gastralgia only by the presence of hæmatemesis. With regard to treatment, cases with obstinate vomiting must be kept in bed on a diet of 1 to 4 ounces of milk and lime water every hour, and the following mixture three times a day: Mag. sulph 40 grains, Ferri sulph. 2 grains, Acid sulph. dil. 15 minims, aq. menth. pip. 1 ounce. If pain is present without vomiting a soda and bismuth mixture is substituted for the above. Patients with hæmatemesis must be fed solely by the rectum until 48 hours have elapsed since the last vomiting of blood. Cure is not complete unless ordinary diet can be taken without discomfort. During convalescence iron must be continued.

About 5 per cent. of all post mortem examinations show gastric ulcer or cicatrix, and of these 40 per cent. present adhesions, so that roughly 2 per cent. of all people have gastric adhesions. These adhesions may, according to Caldwell,[7] interfere with the secretion of gastric juice, and the complete mixing of the food, so giving rise to a distinct form of dyspepsia characterised by the following symptoms: Pain relieved whilst resting or in bed, the pain being of a dragging nature, and increased on exertion, leading to the intuitive avoidance of certain movements, and of heavy meals. The discomfort is relieved by a fairly tight abdominal bandage, and as a rule is not noted until a full half hour after meals. Such cases of dyspepsia due to adhesions may improve in the course of months if the patient be restricted to small, easily-digested meals, the stomach supported by a bandage, and the general health improved, but more frequently operative interference is necessary. A case of this nature, in which gastric dilatation was due to an adhesion formed by the great omentum, is described by Steele in the *British Medical Journal*, February 24th, 1900. Ransom[8] also records a case of pyo-pneumothorax from perforation of a gastric ulcer. The patient died subsequent to operation, and at the post mortem examination the distended stomach was found adherent to liver, diaphragm, and spleen, the small opening of the perforation being in the depths of an indurated mass adherent to the diaphragm and left pleura.

**Cancer of the Stomach.**—The early diagnosis of cancer of the stomach before the presence of a tumour can be detected is a matter of the greatest importance, for authorities are agreed that when once a tumour can be recognised the chances of successful removal by operation are very small. Hemmeter[9] states that in all dyspeptic symptoms there is nothing pathognostic for gastric cancer, but that in early cases diagnosis must rest upon chemical and microscopical examination of the stomach contents and on exploratory incision. During the development of cancer secretion of hydrochloric acid is gradually lost, so that free hydrochloric acid can no longer be detected in the test meals. Parallel with this reduction of acid there is a reduction of pepsin and rennet. Lactic acid is frequently in excess. For purposes of microscopical examination it is necessary to pass an œsophageal tube into the empty stomach, and move it rapidly so as to detach portions of the growth. It may not be possible by this means to detach a fragment giving typical structure of carcinoma, but of almost equal significance is the presence of a large number of cells presenting karyokinetic figures. The Oppler-Boas bacillus, a very long, rod-shaped organism, thick at one end, is constantly present in the stomach contents in malignant cases. In cancer of the stomach the motility and absorbtive power are much impaired. If with the above signs and symptoms a case does not show improvement after three weeks' treatment, exploratory laparotomy is justified. Various observers have estimated that from 25 to 40 per cent. of all fatal cases of cancer are cancer of the stomach. Three-quarters of all cases of gastric cancer occur between the ages of 40 and 70.

[1] Ref. Med., July 4, 1899. [2] Central. f. inn. Med., Aug. 19, 1899. [3] Charlotte Med. Journ., Nov., 1899. [4] La Sem. Médic., Nov. 11., 1899. [5] Amer. Jour. Med. Sci., Aug., 1899. [6] Brit. Med. Jour., Jan. 20, 1899. [7] Brit. Med. Jour., Oct. 28, 1899. [8] Lancet, Nov. 11, 1899. [9] Med. Rec., Oct. 21, 1899.

